# Bilateral muscular slips between superior and inferior rectus muscles: case report with discussion on classification of accessory rectus muscles within the orbit

**DOI:** 10.1007/s00276-018-1976-6

**Published:** 2018-01-24

**Authors:** Robert Haładaj, Grzegorz Wysiadecki, Michał Polguj, Mirosław Topol

**Affiliations:** 1 Department of Normal and Clinical Anatomy, Interfaculty Chair of Anatomy and Histology, Medical University of Lodz, ul. Narutowicza 60, 90-136 Łódź, Poland; 2 Department of Angiology, Interfaculty Chair of Anatomy and Histology, Medical University of Lodz, Łódź, Poland

**Keywords:** Anatomic variation, Extraocular muscles, Inferior rectus muscle, Superior rectus muscle, Orbit

## Abstract

Accessory rectus muscles have rarely been reported as muscular ‘bands’ or ‘slips’ originating from the common tendinous ring (annulus of Zinn) and inserting in atypical location. This group of muscles is innervated by the inferior branch of the oculomotor nerve, lies on lateral side of the optic nerve and inserts in rectus muscles. Since there are only few descriptions of such unusual findings in the medical literature, the anatomical data on accessory rectus muscles is limited. Furthermore, existing reports vary in terms of studied objects (cadavers or living subjects), medical history (absence or presence of ocular movement disorders or eye movement abnormalities) and details of anatomical description. This report complements earlier publications and provides complete anatomical description of the accessory rectus muscle observed bilaterally during the dissection of a 68-year-old male cadaver with no eye movement abnormalities reported in the medical history. The accessory rectus muscle was divided into two ‘slips’ or ‘heads’—superior and inferior—running in the sagittal plane (laterally to the optic nerve and the main trunk of the ophthalmic artery) and attached to the superior and inferior rectus muscles. Noticeable thickening of both superior and inferior rectus muscles at the insertion point of the accessory muscle heads was observed only in the sagittal plane. On both sides, the inferior head of the accessory rectus muscle was innervated by one of sub-branches derived from the inferior branch of the oculomotor nerve. No sub-branches to the superior head were macroscopically observed during the dissection. The classification, embryological background and clinical relevance of this variation have been discussed.

## Introduction

Among seven skeletal extraocular muscles (EOMs), four recti (superior, inferior, medial and lateral) and two oblique (superior and inferior) muscles control movements of the eye, whereas levator palpebrae superioris controls upper eyelid elevation [14]. The four rectus muscles are arranged like a cone with the origins located at the common tendinous ring (annulus of Zinn). Certain nerves (including optic, oculomotor and abducens nerves) and vessels, ciliary ganglion, orbital pad of fat, as well as greater part of the eyeball lie within the cone. Anatomical variations of EOMs, however rare, include: absence of certain muscles, duplication or occurrence of additional bellies, occurrence of slips or bridges between selected muscles, as well as anomalies of innervation or insertions [1, 10]. Such variations may cause a number of changes in morphology and spatial organization of structures located within the orbit. That may be of importance for orbital imaging or during surgical procedures performed on EOMs [7, 8, 15, 17].

Accessory rectus muscles have rarely been reported as muscular bands or slips originating from the common tendinous ring and inserting in atypical location. The muscles from this group are innervated by the inferior branch of the oculomotor nerve, lie on lateral side of the optic nerve and are attached to rectus muscles. There are only few descriptions of similar unusual findings [6–8, 16–18]. However, existing reports vary in terms of studied objects (cadavers or living subjects), medical history (absence or presence of ocular movement disorders or eye movement abnormalities) and details of anatomical description.

The aim of this case report was to present a complete anatomical description of accessory rectus muscles, including: medical history of the body donor, detailed report on observations performed during dissection of the orbital structures, as well as discussion on embryological background and clinical significance of the observed variation.

## Case description

A 68-year-old male cadaver was subjected to routine dissection for scientific and teaching purposes. No head injuries, surgical interventions within the head or eye movement abnormalities were reported in the medical history of the body donor. After eyelid elevation normal rest position of the eyes was observed on both sides.

The skull was opened according to the previously described protocol [19, 20]. After removal of the brain, the entire superior wall and the large part of lateral wall of the orbit were removed on both sides using Luer bone rongeur and bone chisel. The superior orbital fissure and the optic canal were also opened. The orbital fascia and the attachment of the inferior oblique muscle were bluntly separated from the bones and all content of the orbit was harvested en bloc. Further stages of dissection were performed at 2.5× magnification obtained with HEINE HR 2.5× High Resolution Binocular Loupe (HEINE Optotechnik GmbH & Co. KG, Herrsching, Germany). The measurements were made in situ with a Digimatic digital caliper (Mitutoyo Company, Kawasaki-shi, Kanagawa, Japan).

A detailed anatomical investigation of EOMs was assessed on both sides. The orbital fascia was carefully removed. The levator palpebrae superioris and the superior oblique muscles showed normal morphology. When the insertion of the lateral rectus muscle (LR) had been cut and the muscle was freed along its entire length, the access to the deeply located structures was gained. Both the LR and the territory of the abducens nerve were normal. At this stage of the procedure, bilateral presence of unusual muscular structure spanning vertically between superior and inferior rectus muscles was revealed. These rare anatomical findings were recognized as the accessory (supernumerary) rectus muscles.

**Fig. 1:**
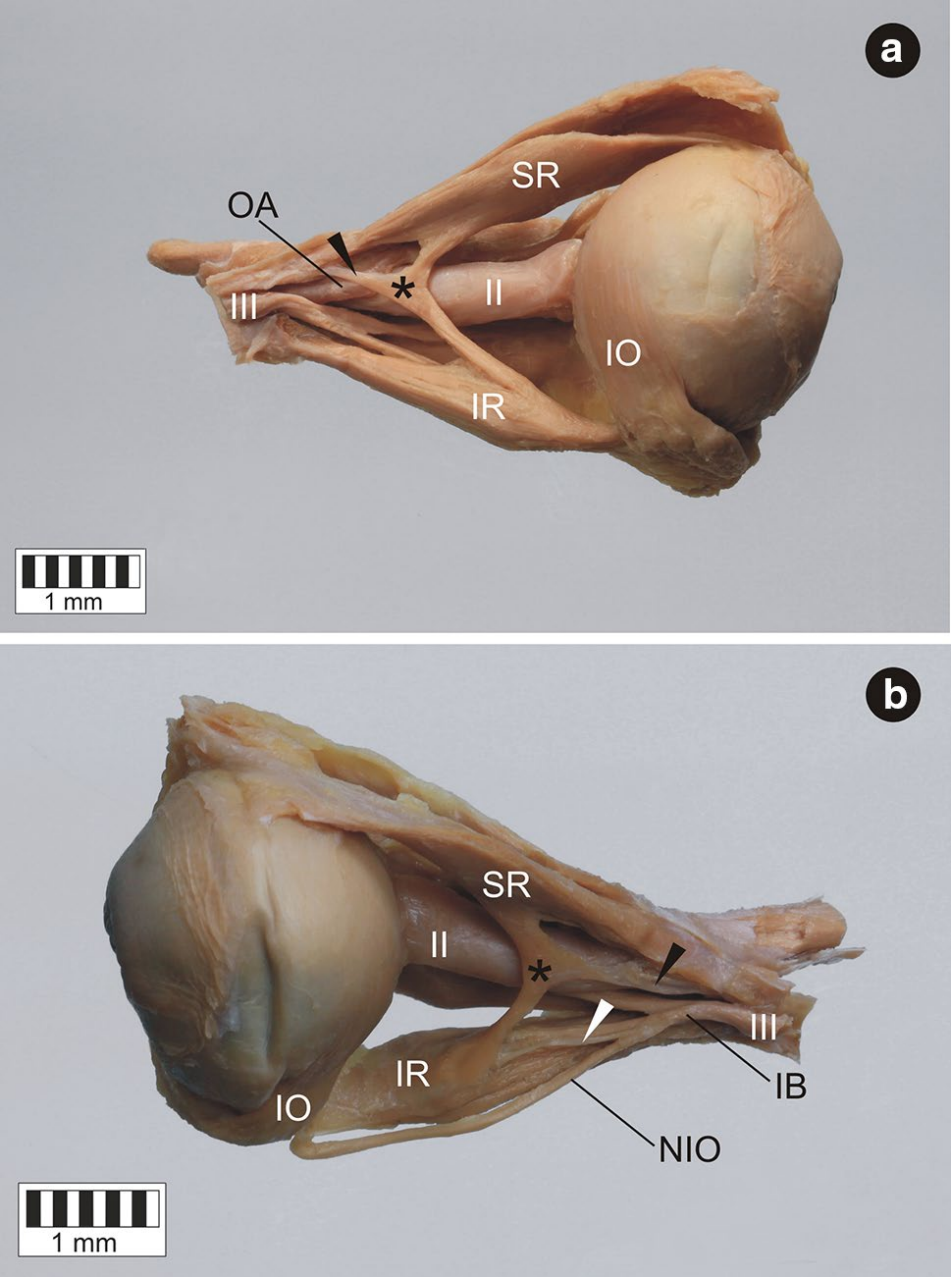
Atypical muscular slips between superior and inferior rectus muscles. Lateral view. The atypical muscle (marked by black asterisk) originates at the common tendinous ring (origin marked by black arrowhead) and passes laterally to the optic nerve (II) and the ophthalmic artery (OA). **a** Dissection of the specimen harvested from the right orbit. The lateral rectus muscle has been removed to expose the accessory muscle. **b** Dissection of the specimen harvested from the left orbit. One of sub-branches derived from the inferior branch of the oculomotor nerve (IB) innervates inferior slip of the accessory muscle (this small branch has been marked by white arrowhead). *IR* inferior rectus muscle, *IO* inferior oblique muscle, *NIO* nerve to inferior oblique, *SR* superior rectus muscle, *III* oculomotor nerve

**Fig. 2:**
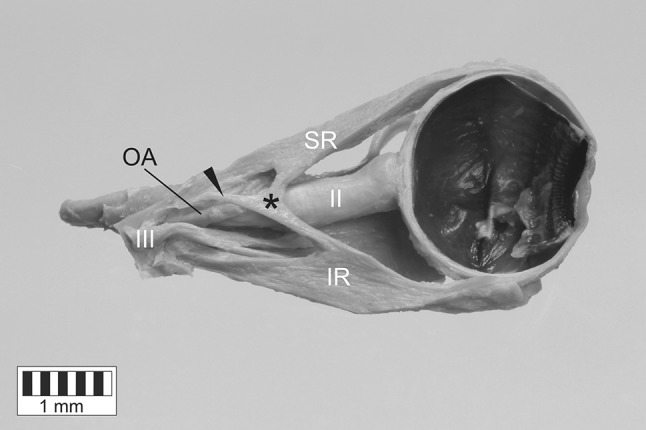
Two slips (heads) of the accessory muscle fusing with the superior and inferior rectus muscles. Sagittal section of the specimen harvested from the right orbit. Lateral view. Thickening of both superior and inferior rectus muscles at the insertion point of the accessory muscle slips has been clearly visualized. Black asterisk marks the accessory muscle, black arrowhead marks the origin of the accessory muscle located at the common tendinous ring, *II* optic nerve, *III* oculomotor nerve, *IR* inferior rectus muscle, *SR* superior rectus muscle, *OA* ophthalmic artery

On both right and left side, the short tendon (of 7.1 mm length on the right and 9.8 mm length on the left side) of the atypical muscle originated at the common tendinous ring and passed laterally to the optic nerve and the ophthalmic artery. The ciliary ganglion was located between the accessory rectus and lateral rectus muscles. Both right and left accessory rectus muscle was divided into two ‘slips’ or ‘heads’—superior and inferior—running in the sagittal plane and attached to the superior (SR) and inferior rectus (IR), respectively (Fig. 1). The dimensions of both heads of the supernumerary muscles are presented in Table 1. The width of the supernumerary muscle measured at the junction of its both heads was 1.02 on the right side and 0.89 on the left side. Noticeable thickening of both SR and IR at the insertion point of the accessory muscle slips was observed only in the sagittal plane (Fig. 2). The maximal thickening (maximal distance between superior and inferior surface) of SR was 4.63 mm on the right and 5.22 on the left side (the width of SR measured at this point was 9.21 mm on the right and 9.36 mm on the left side). The maximal thickening of IR was 6.97 mm on the right and 6.03 mm on the left side (the width of IR measured at this point was 7.22 mm on the right and 8.09 mm on the left side).

**Table 1:** Selected dimensions of both heads of accessory rectus muscles observed in this study

	Selected dimensions of the superior head of the accessory rectus muscle(mm)		Selected dimensions of the inferior head of the accessory rectus muscle (mm)	
	Width at the insertion to SR	Width in the middle part	Width at the insertion to IR	Width in the middle part
Right side	2.2	0.9	2.5	1.5
Left side	2.4	1.1	2.2	1.4

	OAR-ISHAR	ISHAR-ISR	OAR-IIHAR	IIHAR-IIR
Right side	13.7	22.1	20.2	17.6
Left side	17.5	20	19.2	18.2

*OAR-ISHAR* distance from origin of accessory rectus muscle to insertion of superior head of accessory rectus muscle, *ISHAR-ISR* distance from insertion of superior head of accessory rectus muscle to insertion of superior rectus muscle, *OAR-IIHAR* distance from origin of accessory rectus muscle to insertion of inferior head of accessory rectus muscle, *IIHAR-IIR* distance from insertion of inferior head of accessory rectus muscle to insertion of inferior rectus muscle

Both right and left accessory rectus muscle was innervated by the inferior branch of the oculomotor nerve. One of sub-branches derived from the inferior branch of the oculomotor nerve supplied inferior head of the accessory muscle near its insertion to the IR (Fig. 1b). No sub-branches to the superior head were macroscopically observed during the dissection. The diameter of the right oculomotor nerve, its inferior branch and sub-branch to the inferior head of the accessory rectus was 1.46, 0.99, and 0.31 mm, respectively. The same diameters measured on the specimen taken from the left side were 1.4, 1.05, and 0.38 mm, respectively.

**Fig. 3:**
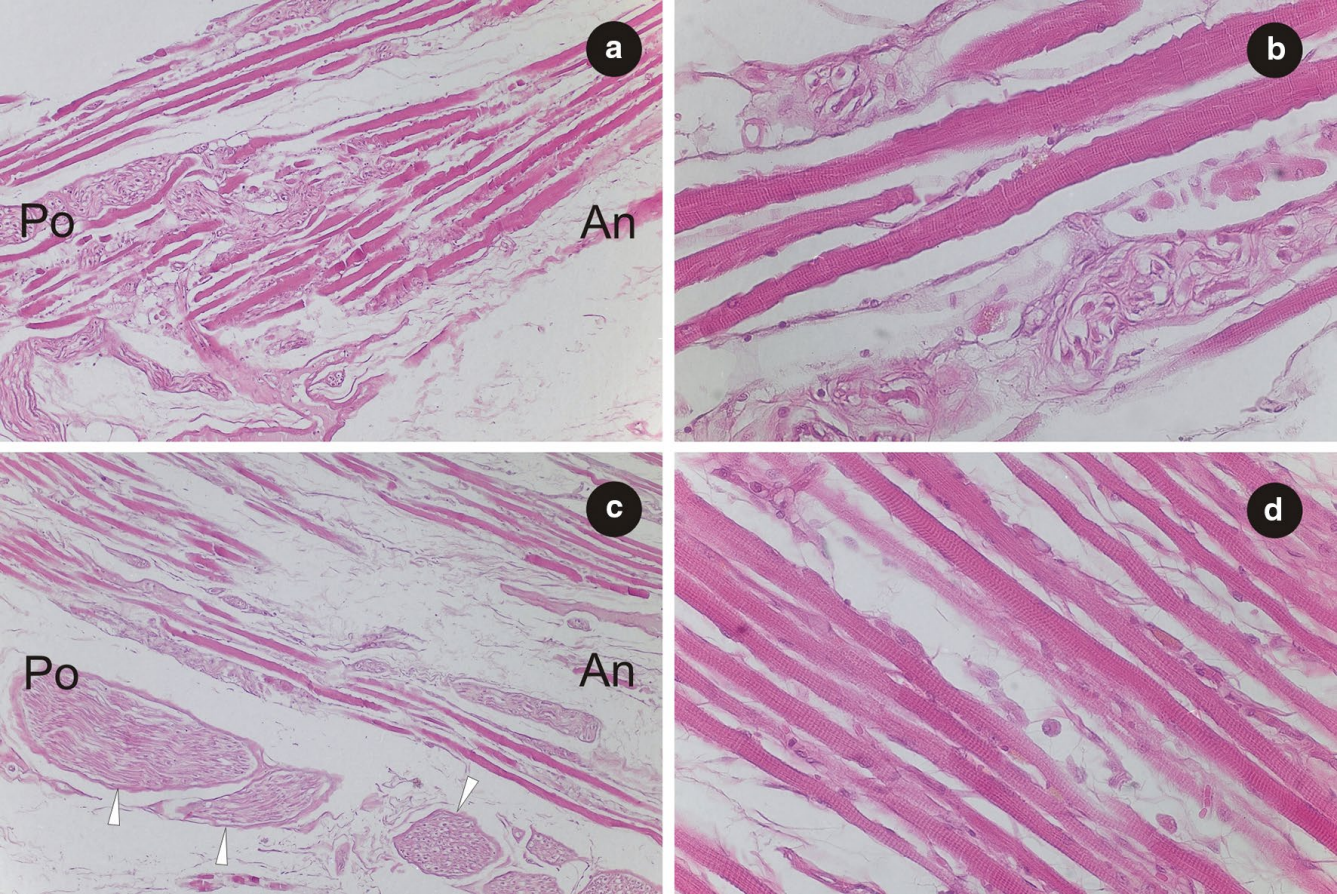
Histological observations on the right accessory rectus muscle. **a** Part of the superior head of the right accessory rectus muscle. Fibers of striated skeletal muscle have been visualized. H&E stain, ×10 objective. **b** Sample fibers of striated skeletal muscle obtained from the superior head of the right accessory rectus muscle. The striations of skeletal muscle tissue have been visualized. H&E stain, ×40 objective. **c** Part of inferior head of the right accessory rectus muscle shown near its origin from the inferior rectus muscle. Fibers of striated skeletal muscle as well as cross sections of small nerves (marked by white arrowheads) have been visualized. H&E stain, ×10 objective. **d** Sample fibers of striated skeletal muscle obtained from the inferior head of the right accessory rectus muscle. The striations of skeletal muscle tissue have been visualized. H&E stain, ×40 objective. *An* anterior, *Po* posterior

**Fig. 4:**
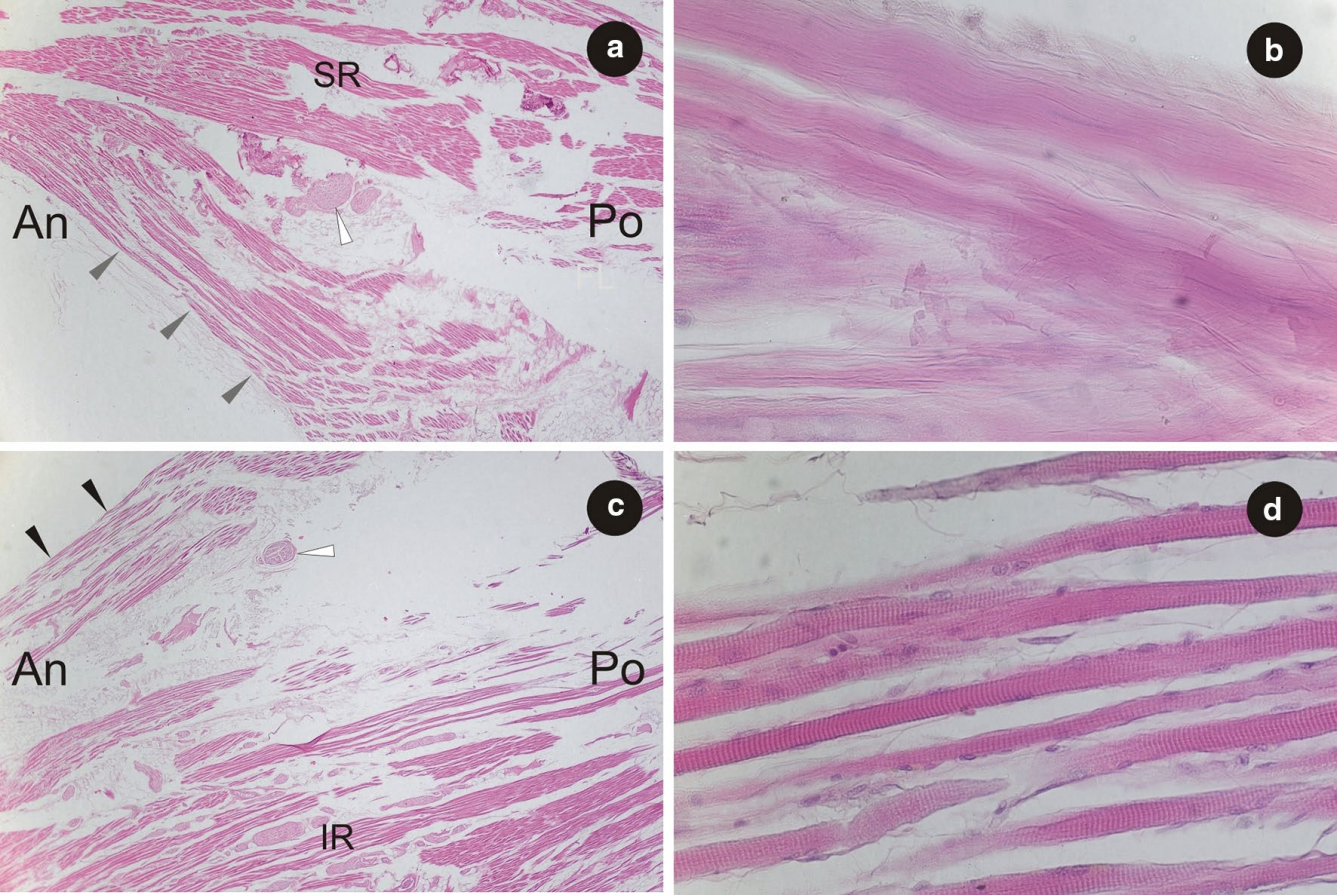
Histological observations on the left accessory rectus muscle. **a** Part of the superior head of the left accessory rectus muscle (marked by grey arrowheads) shown at its origin from superior rectus muscle (SR). H&E stain, ×2 objective. Fibers of striated skeletal muscle and cross-section of small nerve (marked by white arrowhead) have been visualized. **b** Part of the tendon with visible bundles of collagen fibers (tissue sample taken from the origin of the left accessory rectus muscle). H&E stain, ×40 objective. **c** Part of inferior head of the left accessory rectus muscle (marked by black arrowheads) shown at its origin from the inferior rectus muscle (IR). H&E stain, ×2 objective. Fibers of striated skeletal muscle and cross section of small nerve (marked by white arrowhead) have been visualized. **d** Sample fibers of striated skeletal muscle obtained from the inferior head of the left accessory rectus muscle. The striations of skeletal muscle tissue have been visualized. H&E stain, ×40 objective. *An* anterior, *Po* posterior

To supplement the research, both (right and left) accessory rectus muscles were subjected to histological examination. The classical paraffin method was used to obtain serial sections (each about 7 µm in thickness). Then, standard hematoxylin and eosin (H&E) staining was performed. The samples obtained in this way were evaluated under biological microscope (OLYMPUS CX43) with installed camera. The presence of striated skeletal muscle tissue was confirmed in the superior and inferior head of both right and left accessory rectus muscle (Figs. 3, 4). The presence of bands of tendinous tissue was observed in tissue samples taken from the origins of both accessory rectus muscles (compare Figs. 1, 2, 4b).

## Discussion

Anomalous muscular bands occurring in the orbit are rare conditions. This group of variations manifests as muscular slips between certain orbital structures such as: common tendinous ring, EOMs and the globe. Kightlinger et al. [8] distinguished three types of ‘orbital bands’: anomalous muscular bridges connecting two muscles; bands of fibrous tissue adjacent to the muscles and attached to the globe; and muscles that arise from the posterior orbit and have insertions at the globe or at the extraocular muscles. Orbital bands may include both acquired (posttraumatic or postsurgical) and congenital conditions [8]. Congenital conditions may be classified in a structural basis. According to their morphology and innervation, anomalous muscular structures located within the orbit can be assigned into two categories: retractor bulbi muscles and accessory (or supernumerary) rectus muscles [6, 16].

EOMs of vertebrates develop from two populations of mesenchymal cells [2]. Paraxial head mesoderm contributes to forming of lateral rectus and superior oblique muscle. These two muscles are characterized by specific innervation by the abducens and trochlear nerve, respectively. The other muscles (superior, inferior and medial rectus, as well as inferior oblique) are derived from prechordal head mesoderm and innervated by the oculomotor nerve. Embryological background of accessory rectus muscles may be explained by disturbances in differentiation of the superior and inferior mesenchymal/mesectodermal complexes [2]. As shown in previous studies, neuronal precursors demonstrate strictly defined specificity (target organ) [5, 20]. The study of Michalak et al. [13] also suggests that EOMs are required for terminal branching of developing cranial nerves. Based on the knowledge of morphogenesis and development of innervation, it may be presumed, that the accessory rectus muscles, innervated by the inferior branch of the oculomotor nerve, may be developmentally nearer to the inferior mesenchymal/mesectodermal complex.

In early descriptions, anomalies of the orbital muscles in humans were considered to be remnants of the retractor bulbi muscle [18]. Retractor bulbi typically consists of four slips that surround the optic nerve. It is innervated mainly by the abducens nerve. However, retractor bulbi responses to oculomotor nerve and nucleus stimulation have been suggested in cat [12]. Such double innervation pattern may allow the oculomotor retractor bulbi motor units to be activated during different types of eye movements. Retractor bulbi muscle occurs in amphibians, reptiles and most mammals [18]. According to recent studies, retractor bulbi group (actually, remnants of retractor bulbi) in humans involves muscles innervated mainly by the abducens nerve and may be represented by: muscular slip arising from lateral rectus muscle; single muscle bundle between lateral and superior or middle rectus muscles; or muscular slips arising from common tendinous ring and attached to posterior surface of the globe [1].

**Fig. 5:**
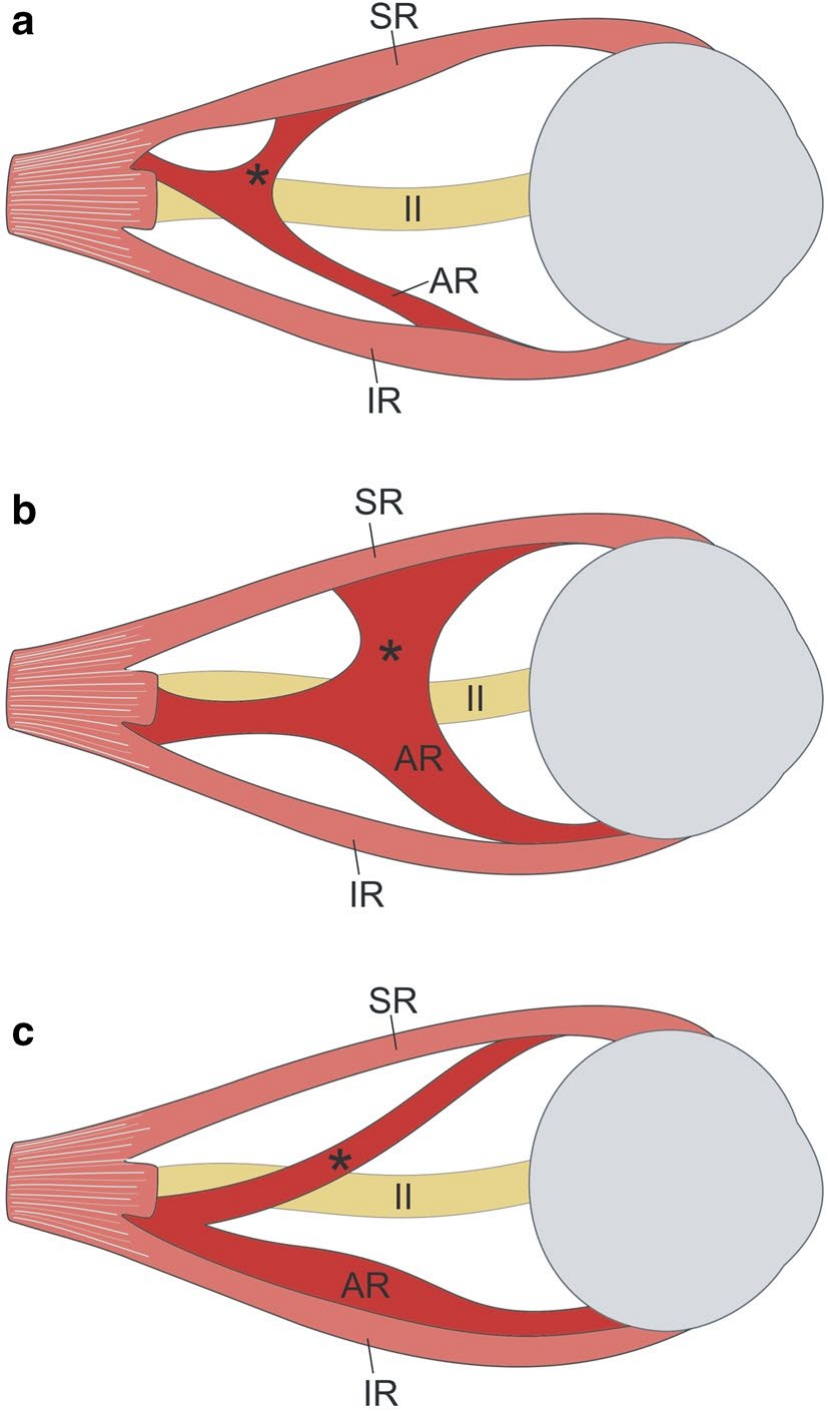
Different variants of the supernumerary orbital muscles reported in the literature. Lateral view. For ease of comparison and increased transparency, the same side has been presented on all schemes. Terminology applied by von Lüdinghausen et al. [17] has been taken into account. **a** Anatomical variation described in this report—an accessory muscle observed on 68-year-old cadaver with no eye movement abnormalities reported in the medical history. The accessory muscle is divided into two delicate slips (heads): superior (marked by black asterisk)—forming muscular bridge connected to the superior rectus muscle (SR); and inferior—corresponding to accessory rectus muscle (AR) and attached in the anterior half of the inferior rectus muscle (IR). **b** Supernumerary orbital muscle (AR) with a broad muscular bridge (marked by black asterisk) to the SR and attachment to the anterior part of the IR. The accessory muscle was well-separated from the IR. **c** Supernumerary orbital muscle (AR) with a thin muscular bridge (marked by black asterisk) to the SR and close attachment to the anterior part of the IR. Variants b and c were described by von Lüdinghausen et al. [17] on the adult cadaver with no problems with mobility of the eyeball in the medical history. *II* optic nerve

Accessory (or supernumerary) rectus muscles seem to be a more homogeneous group. As previously mentioned, the common feature of this group is an origin at the annular tendon (annulus od Zinn), localization lateral to the optic nerve and innervation by the inferior branch of the oculomotor nerve. However, a limited amount of anatomical studies is currently available on this issue. Kakizaki et al. [6] described bilateral anomalous muscle linking superior and inferior rectus muscles in the orbit of 45-year-old female cadaver with no ocular movement disorders in the medical history. It was the first report of such finding in a representative of the Asian population. The case reported by Kakizaki et al. [6] seems to be similar to our description. However, opposed to our observations, Kakizaki et al. detected no definite nerve insertion in the accessory rectus muscles [6]. Like in the presented case (Figs. 1, 2, 5a), no other abnormalities were found within the orbit. A case of bilateral existence of the accessory rectus muscles was also described by von Lüdinghausen [16, 17]. Von Lüdinghausen’s report [17] was also based on dissection of the adult cadaver with no problems with mobility of the eyeball. In the cited publication, on the right side, there was detected a supernumerary orbital muscle with a broad (4 mm) muscular bridge to the SR and attachment to the anterior part of the IR (Fig. 5b). The accessory muscle was well-separated from the IR. On the left side, a different arrangement was observed. The supernumerary orbital muscle also sent a thin (2 mm) muscular bridge to the SR. Nevertheless, in contrast to the right side, the muscular bridge running to the SR originated in the region of the common tendinous ring, while the accessory rectus muscle showed close attachment to the anterior part of the IR (Fig. 5c).

**Fig. 6:**
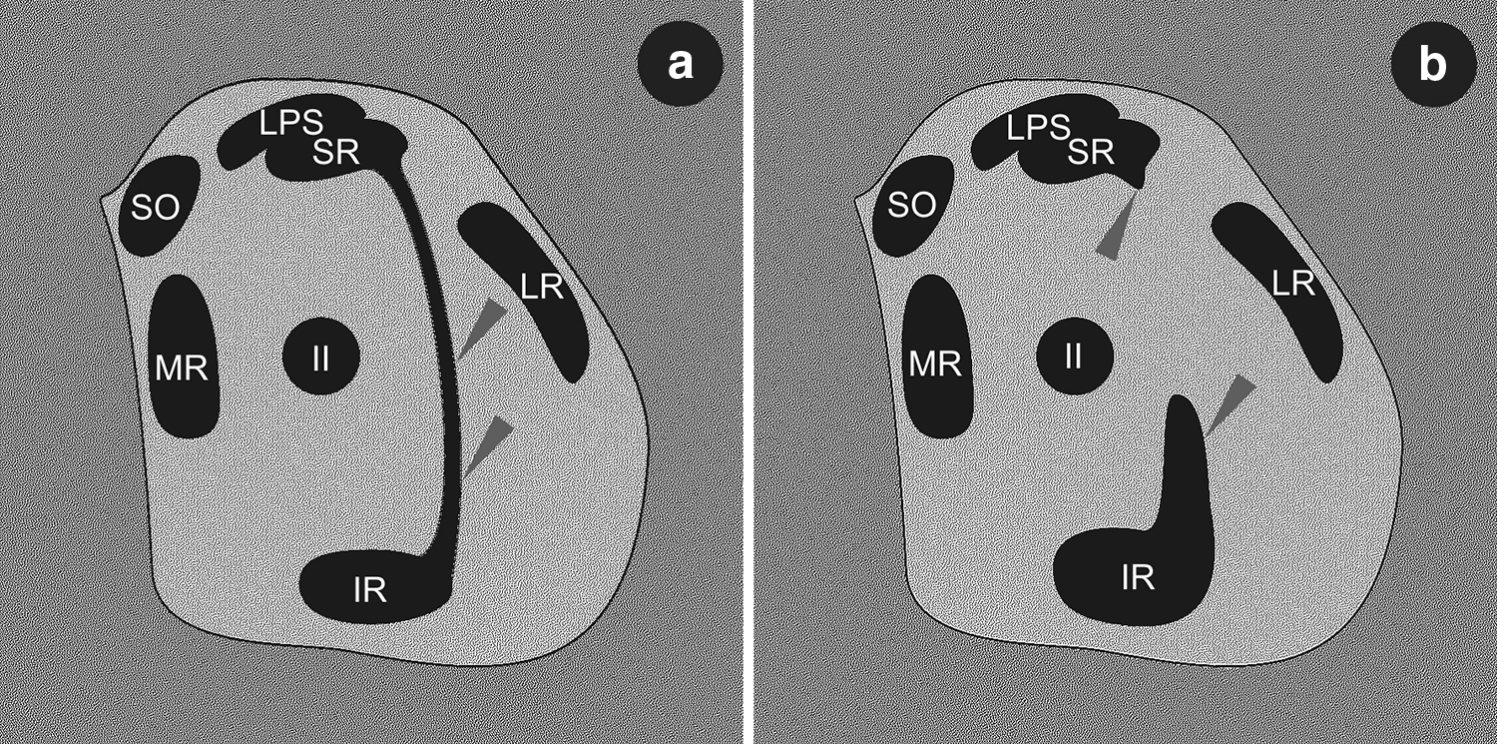
Schematic drawings simulating MRI or CT coronal scans demonstrating spatial organization of muscular structures within the orbit in the event of presence of accessory (supernumerary) rectus muscles or muscular bands between superior and inferior rectus muscles. The drawings have been prepared on the basis of comparison of different MRI scans presented by Khitri and Dremer [7] and Kightlinger at al. [8]. **a** Complete muscular bridge seen between temporal edges of superior and inferior rectus muscles (marked by grey arrowheads). On drawing (**b**) only fragments of certain heads of the supernumerary rectus were captured (grey arrowheads). *IR* inferior rectus muscle, *LR* lateral rectus muscle, *LPS* levator palpebrae superioris muscle, *MR* medial rectus muscle, *SR* superior rectus, *SO* superior oblique muscle, *II* optic nerve

Muscular bands or bridges between SR and IR have also been observed during imaging studies (Fig. 6). Orbital imaging, including high-resolution MRI, have played a special role in deepening the knowledge of the morphology and function of the EOMs and their associated connective tissues [4]. Magnetic resonance imaging of tissues compatible with supernumerary extraocular muscles was studied by Khitri and Demer [7]. These authors provided the largest case series of supernumerary human EOMs identified on high resolution MRI. They assessed incidence of all variants of orbital bands at 0.8% among 118 orthotropic subjects and at 2.4% among 453 patients with strabismus [7]. In the same study, the superior–inferior rectus bands were seen only in 33% of all types of bands detected within the orbit [7]. A case series of orbital bands connecting the SR to the IR was presented by Kightlinger et al. [8]. These authors described a series of seven cases with muscular bands that connected the temporal edges of the SR and IR.

According to Kightlinger et al. [8] the clinical significance of muscular bands or slips located within the orbit ‘is uncertain and possibly depends on size and location’. Such atypical structures are often found accidentally, as they do not always produce eye movement disorders. The presence of accessory rectus muscles may be asymptomatic, what has been confirmed both in our report and in previous studies [6, 16, 17]. However, muscular bands coursing under the optic nerve and connecting the horizontal rectus muscles may be the probable cause of restrictive strabismus [7]. Accessory muscular bands located within the orbit may also be a component of ocular congenital cranial dysinnervation disorders (CCDDs) [3]. Furthermore, orbital bands may be confused with normal vessels (arteries or veins) or with pathologies located within the orbit (lymphoma, orbital pseudotumor, vascular malformations, sarcoid, or metastasis) [8]. Thus, knowledge of normal anatomy and variations of EOMs may be clinically important for orbital imaging, differential diagnostics of eye movement disorders, as well as for surgical procedures involving this group of muscles [7–11, 15–17].

## Conclusions

Accessory rectus muscles in the orbit can be defined, on the basis of their anatomy, as the muscular structures originating at the common tendinous ring, running vertically (lateral to the optic nerve) and inserting in rectus muscles. They are innervated by the inferior branch of the oculomotor nerve. Such rare findings may be relevant during orbital imaging or EOMs surgery.
